# Comparison between Prothrombin Complex Concentrate (PCC) and Fresh Frozen Plasma (FFP) for the Urgent Reversal of Warfarin in Patients with Mechanical Heart Valves in a Tertiary Care Cardiac Center

**Published:** 2015

**Authors:** Bahram Fariborz Farsad, Reza Golpira, Hamideh Najafi, Ziae Totonchi, Shirin Salajegheh, Hooman Bakhshandeh, Farshad Hashemian

**Affiliations:** a *Rajaie Cardiovascular, Medical and Research Center, Iran University of Medical Sciences, Tehran, Iran.*; b *Department of Clinical Pharmacy, Pharmaceutical Sciences Branch, Islamic Azad University.*

**Keywords:** Prothrombin complex concentrates, Fresh frozen plasma, Warfarin reversal

## Abstract

Fresh frozen plasma (FFP) and prothrombin complex concentrate (PCC) reverse oral anticoagulants such as Warfarin. We compared the standard dosage of FFP and PCC in terms of efficacy and safety for patients with mechanical heart valves undergoing interventional procedures while receiving Warfarin. Fifty patients were randomized (25 for each group) with mechanical heart valves [international normalized ratio (INR) >2.5]. FFP dosage was administered based on body weight (10-15 mL/Kg), while PCC dosage was administered based on both body weight and target INR. INR measurements were obtained at different time after PCC and FFP infusion. The mean ± SD of INR pre treatment was not significantly different between the PCC and FFP groups. However, over a 48-hour period following the administration of PCC and FFP, 76% of the patients in the PCC group and only 20% of the patients in the FFP group reached the INR target. Five (20%) patients in the PCC group received an additional dose of PCC, whereas 17 (68%) patients in the FFP group received a further dose of FFP (*P*=0.001). There was no significant difference between the two groups in Hb and Hct before and during a 48-hour period after PCC and FFP infusion. As regards safety monitoring and adverse drug reaction screening in the FFP group, the INR was high (INR > 2.5) in 86% of the patients. There was no report of hemorrhage in both groups. PCC reverses anticoagulation both effectively and safely while having the advantage of obviating the need to extra doses.

## Introduction

Every year, approximately 300,000 patients undergo heart valve surgery worldwide(1). Although mechanical heart valves have the advantage of a superior durability over bioprosthetic valves, they suffer from much higher and persistent risk of thromboembolism, requiring lifelong anticoagulation therapy. Vitamin K antagonists such as Warfarin are the existing long-term standards for anticoagulation in patients having had cardiac valve replacement (2). The risk of Warfarin-induced bleeding complications is well known and is typically managed with vitamin K, which will restore the production of vitamin K-dependent coagulation factors within 12-24 hours. It is not, however, always feasible to wait for endogenous restoration of coagulation factors, especially in patients scheduled for either urgent or semi-urgent coronary artery bypass grafting surgery (CABG) involving cardiopulmonary bypass (CPB) or valvular surgery in which coumarin effects should be reversed rapidly.

Fresh frozen plasma (FFP) and prothrombin complex concentrate (PCC) are currently deemed suitable for increasing the concentration of vitamin K-dependent coagulation factors. These two products contain factors II, VII, IX, and X and thus help restore normal levels of the clotting fraction (3,4).

FFP is human plasma frozen within a specific time period after collection both from a donor (5). The risks of FFP include disease transmission, anaphylactoid reactions, alloimmunization, excessive intravascular volume, transfusion-related acute lung injury, and increased risk of infections (6-9).

PCC is derived from pooled, virus-inactivated human plasma products and is known to provide coagulation factors appropriately and rapidly. The majority of PCC products contain vitamin K-dependent coagulation factors (II [prothrombin],VII, IX, and X), as well as therapeutically effective concentrations of thrombo inhibitors (protein C and S)(10,11). The early PCC composition contained the three coagulation factors of II, IX and X, with no or very little amount of factor VII. Nevertheless, newer formulations, available in some European countries, include normal amounts of factor VII and are thus known as “four-factor concentrates” or “4-factor PCCs” (12).

In this randomized study, we compared the efficacy of the infusion of PCC and FFP in adult patients with mechanical heart valves who were undergoing Warfarin therapy and required Warfarin reversal.

## Experimental

*Study population*

This study was conducted in compliance with the Helsinki Declaration. The protocol was submitted for approval to the Ethics Committee of Rajaie Cardiovascular, Medical and Research Center, an Iranian tertiary health care hospital. Fifty adult patients with mechanical heart valves who were admitted to the hospital and provided written informed consent were enrolled in the present study between July 2012 and February 2013. All of the patients were undergoing interventional procedures while receiving Warfarin therapy.

*Inclusion and exclusion criteria*

The inclusion criteria comprised weight under100 Kg, the age between 18-85 years old, Warfarin consumption prior to intervention, initial international normalized ratio (INR) > 2.5, and consumption of FFP or PCC. The exclusion criteria were comprised of renal or hepatic insufficiency, allergic reaction to blood products, past history of Heparin-induced thrombocytopenia and disseminated intravascular coagulation, active thrombosis or pulmonary embolism, intracardiac thrombus, and pregnancy or breastfeeding.

*Treatment*

Two treatment groups for the reversal of increased INR levels were defined: patients treated with PCC (n=25) and those treated with FFP (n=25).Vitamin K was never used for the patients in either group.

In the PCC group, all the patients were given PCC (Uman Complex D.I.) (Kedrion, Castelvecchio Pascoli, Italy), which is a 3-factor PCC, including factor II, factor IX, and factor X. Uman Complex is a lyophilized PCC, which is purified human plasma through two ion-exchange chromatography steps. Each vial of Uman Complex contains a relatively high concentration of coagulation factor II (25 IU⁄mL), factor IX (25 IU⁄mL), factor X (20 IU⁄mL), Heparin (< 12.5 IU/mL), and antithrombin (< 0.125 IU/mL). The PCC administration was based on the patients’ body weight (in accordance with relevant INR), initial INR, and target INR in keeping with the manufacturer’s prescribed individualized dosing regimen (Table 1). If the INR was high after the initial PCC administration, an additional dose was administered.

In the FFP group, FFP was administered based on the patients' body weight (10-15 mL/Kg). If the INR was still high following the initial FFP administration, additional FFP (JMS Singapore PTE, 300 mL) was administered. The treatment was provided immediately in an emergency setting after block randomization and usually before any INR results were available.

**Table 1:** Recommended uman complex (prothrombin complex concentrate) doses in case of rapid oral anticoagulant therapy reversal *.

**INR value**	2.0-4.0	>4.0 & <6.0
**Dose of PCC to infuse**	25 IU/kg	35 IU/kg

INR, international normalized ratio; PCC, prothrombin complex concentrate

* Kedrion Manufacturing Company's recommendation package (insert 2010)

*Study outcomes*

INR measurements were obtained 30 minutes after the infusion of FFP and PCC. In both groups, serial INR measurements were obtained at 4, 8, 12, 16, 20, 24, and 48 hours post PCC and FFP infusion. The groups were compared on the basis of demographics, units of FFP, units of PCC, number of patients achieving INR < 2.5, number of patients requiring extra dose(s) of each drug to reach the target INR, and adverse drug reaction (based on the Kedrion assay of adverse drug reaction*) to FFP and PCC. 

*Laboratory and clinical assessment*

Blood samples were collected for the determination of INR prior to infusion and at intervals of 0.5, 4, 8, 12, 16, 20, 24, and 48 hours as well. Hemoglobin (Hb) and Hematocrit (Hct) were determined at baseline and after 48 hours. 

The INR and hematology parameters were measured at the laboratory of the study center. At enrolment time, all the patients underwent a complete clinical assessment, encompassing medical history, physical examination, and determination of vital signs.

The occurrence of any adverse events, including death, thromboembolic complications, and allergic reactions, was monitored.

*Statistical analysis*

The data are presented as mean ± standard deviation (SD) for the interval variables and count (%) for the categorical variables. The one-sample Kolmogorov-Smirnov test was applied to investigate the fitness of the interval data to normal distribution. The clinical outcomes were compared between the two groups using the Student t-test or the Pearson chi-squared test and repeated measure analysis of variance (ANOVA) models. A *P* ≤ 0.05 was considered statistically significant. SPSS® 15 for Windows® (SPSS Inc., Chicago, Illinois) was utilized for statistical analysis.

## Results

Between July 2012 and February 2013, a total of 50 patients were included and randomized into the 3-factor PCC group (n =25) (administered according to the INR) and the FFP group (n = 25) (10-15 mL/Kg).

Out of the 50 patients recruited in our study, 35 patients were admitted to the Intensive Care Unit (ICU) and 15 patients to the Coronary Care Unit (CCU). The baseline characteristics and demographic data of the study population are summarized in Table 2. The overall mean (standard deviations (SD)) age was 66.73 ± 7.81 years, and 56% of the patients were male. Patient characteristics did not differ between the two groups. All the patients had a history of cardiovascular disease, hypertension, and valvular surgery. All the patients were under treatment with Warfarin sodium.

**Table 2:** Baseline demographic and clinical data of the study population

All patients			**PCC**	**FFP**	***P*** **-value**
Gender (n;%)					
	Male	28 (56)	10 (40%)	18 (72%)	0.73
	Female	22 (44)	15 (60%)	7 (28%)	0.74
Age			66.73±7.81	65.8±6.79	0.70
Weight			68.73±9.37	67.47±10.07	0.469
Height			164.77±6.57	161.73±6.46	<0.01
Heart rate			97.60±11.08	96.93±10.39	0.75
Systolic BP			119.87±15.10	123.33±15.88	0.22
Diastolic BP			72.90±10.26	71.47±11.95	0.46
EF			47.50±5.21	48.67±5.16	0.23
Temperature			36.77±0.96	36.93±0.821	019
SGOT			23.60±10.72	24.73±11.47	0.57
SGPT			17.27±11.47	17.07±10.36	093
Creatinine			1.08±0.29	1.14±0.32	0.31

Data are presented as mean ± SD.Standard Deviation

PCC, prothrombin complex concentrate; FFP, fresh frozen plasma; BP, blood pressure; EF, ejection fraction; SGOT, serum glutamic oxaloacetic transaminase; SGPT, serum glutamic pyruvic transaminase

The types of cardiac surgery in the valvular patients are summarized in Table 3.

**Table 3:** The types of cardiac surgery in the valvular patients and distributions in patients in the PCC and FFP groups

**Type of valve operation**	**FFP **(n=25)	**PCC **(n=25)
MVR	3 (12%)	6 (24%)
MVr	4 (16%)	4 (16%)
AVR	3 (12%)	3 (12%)
Aortic valve repair	4 (16%)	4 (16%)
MVR-AVR	4 (16%)	5 (20%)
TVR-MVR	2 (8%)	1 (4%)
MVR-TVR	2 (8%)	1 (4%)
MVR- AVr- TVR	1 (4%)	0 (0%)
AVR - TVr	2 (8%)	1 (4%)

PCC, prothrombin complex concentrate; FFP, fresh frozen plasma; MVR, mitral valve replacement; AVR, aortic valve replacement; TVR, tricuspid valve repair

The INR values before the infusion of PCC and FFP were not different between the two groups. With respect to efficacy, the INR values after treatment were markedly reduced in the PCC group. The mean (SD) INR pre treatment was 4.02 (1.07) in the PCC group and 4.88 (1.3) in the FFP group. 

The INR was measured 15 minutes as well as 4, 8, 12, 16, 20, 24 and 48 hours after the infusion of PCC and FFP. The mean INR value in the PCC group was markedly reduced compared with that in the FFP group based on serial reporting within 48 hours of administration. The INR values of the PCC and FFP groups are summarized in Table 4. The mean INR value in the PCC group was markedly reduced compared with that in the FFP group based on serial reporting within 48 hours of administration.

**Figure 1:**
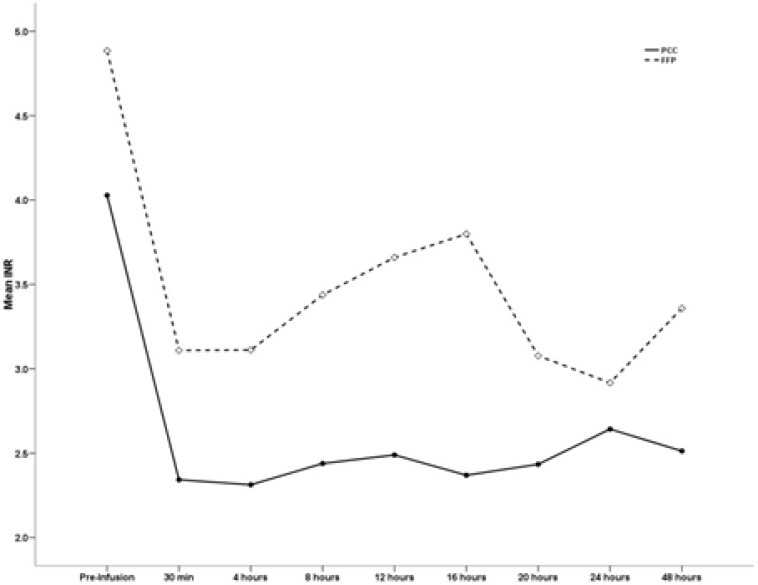
INR fluctuations after PCC & FFP infusion

**Table 4:** INR Variations during the study period

**Time**	**INR (mean ±SD)**		***P*** **-value**
	**PCC **(n=25)	**FFP **(n=25)	
Pre infusion	4.02±1.07	4.88±1.3	0.07
30 min post infusion	2.34±0.7	3.1±1.04	0.03
4 h post infusion	2.31±0.68	3.11±0.83	1. 23
8 h post infusion	2.43±0.68	3.11±0.83	1.34
12 h post infusion	2.48±1.03	3.66±1.35	1. 27
16 h post infusion 3.7±1.71 0.009	2.36±0.87	3.7±1.71	0.01
20 h post infusion	2.43±0.75	3.07±1.18	0.09
24 h post infusion	2.64±1.02	2.91±0.88	0.44
48 h post infusion	2.51±0.84	3.35±0.85	0.01

The proportion of the patients who achieved the target INR was significantly greater in the PCC group (76%) than in the FFP group (20%). The total doses of FFP and PCC as well as the number of the patients achieving INR < 2.5 are demonstrated in Table 5.

**Table 5:** Correlation between prothrombin complex concentrate and fresh frozen plasma dose and reduced target international normalized ratio

**Parameter**	**PCC**	**FFP**
FFP units, mean (SD)	NA	2.6 (0.507)
PCC total dose, units, mean (SD)	2066.6 (0.34)	NA
Patients who achieved INR <2.5, n (%)	19 (76%)	5 (20%)

FFP, fresh frozen plasma; NA, not applicable; PCC, prothrombin complex concentrate

Twenty-two of the patients who required urgent reversal of oral anticoagulation needed additional doses of PCC and FFP to reach the target INR: 5 (20%) patients in the PCC group and 17 (68%) patients in the FFP group. The difference between the two groups was statistically significant (*P* < 005).

**Table 6:** Extra Doses needed for warfarin reversal by PCC & FFP

**Time**	**PCC **(n=25)	**FFP **(n=25)
30 min post infusion	-	-
4 h post infusion	1(4%)	-
8 h post infusion	1(4%)	3(12%)
12 h post infusion	-	5(20%)
16 h post infusion	2(8%)	6(24%)
20 h post infusion	-	3(12%)
24 h post infusion	1(4%)	
48 h post infusion	-	-
Total (*P* value 0.001)	5(20%)	17(68%)

PCC, prothrombin complex concentrate; FFP, fresh frozen plasma.

There was no significant difference between the two groups as regards Hb and Hct before and during a 48-hour period after the infusion of PCC and FFP.

**Table 7:** Hemoglobin and Hematocrit fluctuations following PCC & FFP.

**Hb & Hct Pre & Post Administration**	**PCC **(n=25)	**FFP **(n=25)	**p-value**
Hb pre infusion	9.13±1.22	9.48±1.19	0.44
Hb post infusion	9.64±1.7	10.16±1.32	0.36
Hct pre infusion	29.05±4.7	29.68±4.3	0.71
Hct post infusion	30.98±6.6	32.18±4.4	0.57

Data are presented as mean ± standard deviation.

PCC, prothrombin complex concentrate; FFP, fresh frozen plasma; Hb, hemoglobin; Hct, hematocrit

As for safety monitoring and adverse drug reaction screening, none of the patients treated with either PCC or FFP suffered hemorrhage, although the INR was high (INR > 2.5) in 86% of the FFP group. 

In the FFP group, 68% of the patients needed extra doses. It should be noted that this is not an appropriate choice for patients with mechanical heart valves and may expose them to the risk of volume overload.

## Discussion

The present study is the first randomized head-to-head comparison between 3-factor PCC and FFP as treatment modalities for the reversal of Warfarin-induced anticoagulation in patients with mechanical heart valves who undergo interventional procedures and are at risk of bleeding. The main objective of our study was the assessment of both efficacy (based on the INR) and safety of PCC in patients with mechanical heart valves.

Thromboembolic events are the major cause of morbidity and mortality in patients with mechanical heart valves. Such complications can be decreased by oral anticoagulation after cardiac surgery (13). The fact that these patients are highly susceptible to bleeding under certain interventional procedures such as the insertion of the intravenous (IV) line or the dialysis catheter renders the administration of PCC or FFP in these circumstances advisable. PCC is indicated when rapid correction of prothrombin complex levels is necessary (*e.g*. major bleeding and emergency surgery). In other cases, reducing the dose of vitamin K antagonist and/or administrating vitamin K is usually sufficient (14).

*A) Prothrombin complex concentrate indication in expert guidelines*

The use of PCC has been advocated for the rapid correction of coagulation deficits (15). PCC is yet to be recommended by American guidelines, reviews, or algorithms (16-19), such as the American Heart Association (AHA) or the American College of Cardiology (ACC) either in cardiac or in non-cardiac surgery. The only hint by the AHA is in regard to PCC administration in oral anticoagulant therapy of patients with high INR, in whom PCC has the advantage of causing fewer adverse effects compared with FFP.

However, in European countries, PCC is routinely used in addition to or instead of FFP (19). A case in point is the guideline by the National Health Service (NHS) in 2011, which recommends PCC together with vitamin K if there is bleeding in patients with prosthetic heart valves and high INR. According to the NHS, FFP is indicated if PCC is not available (20). Also, the European Society of Cardiology (ESC) in 2005 published its guideline for valvular heart disease and recommended the use of PCC if bleeding still continues despite FFP (21). Moreover, French guidelines deem PCC superior to FFP in reversing major intraoperative bleeding whether or not vitamin K is administered (22). Along the same lines, the European Medicines Agency’s core summary of the product characteristics for PCC stipulates that treatment of bleeding and perioperative prophylaxis of bleeding in acquired deficiency of the prothrombin complex coagulation factors are indications for the usage of this drug (23). The rationale behind this is that clinicians believe that the level of coagulation in FFP is relatively low and that the volume of FFP required for the correction of coagulopathy is too high (19,24). PCC also offers a number of advantages over FFP, including lower infusion volume, ambient storage, rapid reconstitution, immediate availability, lack of blood group specificity, and better safety profile (25). 

PCC was first introduced into the Iranian Drug Formulary list in 2011 for congenital deficiencies of clotting factors in the prothrombin complex, bleeding episodes in the presence of deficiencies of one or more factors of the prothrombin complex, limitations to the use of FFP because of the risk of circulatory overload or the need for immediate homeostasis, severe liver disease with serious bleeding, preparation for elective surgery carrying the risk of bleeding (*e.g*. liver transplantation),vitamin K deficiency in the presence of life-threatening bleeding, and excessive doses of dicoumarols or the need to suspend them in emergency conditions (*e.g.* acute hemorrhage and urgent surgery).

*B) Efficacy*

PCC is more effective to restore the INR and facilitate coagulation than is FFP. Our results showed that more patients in the PCC group reached the desired INR target than in the FFP group. The mean INR value prior to PCC administration was 4.02 ± 1.07, and the mean INR value before FFP administration was 4.88 ± 1.3. However, the mean INR value decreased to 2.51 during the first 48-hour period after PCC administration, while the mean INR value dropped to 3.35 during the first 48-hour period following FFP administration (*P* = 0.01)

 Mendarte L *et al*. (26) reported that whereas the mean INR value prior to PCC administration was 2.92 (2.54), the mean INR value after PCC was 1.47 (0.44). The mean difference was 1.54 (2.89), which reached statistical significance (*P* = 0.005). Significance was maintained when the general surgery patients were analyzed separately (*P* = 0.001), but that was not the case in the cardiac surgery patients (*P* = 0.36).

Comparative studies have also demonstrated that PCC is more effective than is FFP for correcting patients’ INRs 

*C) Hematocrit and hemoglobin levels*

In our study, there was no significant difference in Hb and Hct between the two groups before and during the first 48-hour period following the infusion of PCC and FFP. In a similar work by Mendarte L *et al*., (26) also there were no significant differences between Hb and Hct before and after PCC.

*D) Adverse drug reactions*

In the present study, no major adverse drug reactions (*e.g*. bleeding) were observed directly in consequence of PCC administration, which can be attributed to the limited sample size of the study. Nonetheless, 68% of the patients treated with FFP needed extra doses; this is not appropriate for patients with valvular heart disease because it may render them vulnerable to volume overload.

Our results demonstrated that PCC conferred more effective reversal of the over coagulated patients than did FFP; this can be due to the high concentration of coagulation factors in PCC. None of the patients treated with either PCC or FFP suffered any active bleeding, although the FFP group patients were at high risk of bleeding because of their high INR levels. 

Thrombotic events have been reported to occasionally complicate PPC infusion, but the differences in the preparations, dosages (correcting the INR to different levels), and patient populations in the available reports make it extremely difficult to quantify the risk. Thrombogenic effects are very likely to vary with different PCC preparations, and there is no evidence in the existing literature to verify a possible association between PCC and thrombosis. 

## Conclusion

In light of our results, PCC is an effective and safe alternative to FFP in patients with mechanical heart valves. We would, therefore, recommend that PCC be employed for rapid and effective correction of Warfarin in patients with mechanical heart valves who require urgent Warfarin reversal. 

The major limitation of the present study is its limited sample size, which may have reduced the power of trial.
